# Physicochemical Properties of Cellulose Nanocrystals Extracted from Postconsumer Polyester/Cotton-Blended Fabrics and Their Effects on PVA Composite Films

**DOI:** 10.3390/polym16111495

**Published:** 2024-05-24

**Authors:** Rivalani Baloyi Baloyi, Bruce Bishop Sithole, Viren Chunilall

**Affiliations:** 1 Department of Chemical Engineering, College of Agriculture, Engineering and Science, University of KwaZulu Natal, Durban 4000, South Africa; sitholeb1@ukzn.ac.za (B.B.S.); vchunilall@csir.co.za (V.C.); 2 Biorefinery Industry Development Facility, Council for Scientific and Industrial Research, Durban 4000, South Africa

**Keywords:** cellulose nanocrystals, postconsumer textile waste, polyester/cotton fabrics, acid hydrolysis

## Abstract

The utilisation of cotton waste as precursors in the synthesis of nanocrystalline cellulose has gained significant attention. This approach suggests a sustainable solution to address the growing concern of textile waste accumulation while simultaneously producing a valuable material. The main aim of this study is to examine the properties of cellulose nanocrystals (CNCs) obtained from postconsumer polyester–cotton waste and assess the effect of different fabric structures on the extraction and these properties. To acquire nanocellulose, a thorough decolourisation pretreatment process was utilised, which involved the treatment of polyester–cotton waste with sodium dithionite and hydrogen peroxide. Consequently, the postconsumer material was then treated with an acid hydrolysis method employing a 64% (*v*/*v*) sulphuric acid solution at 50 °C for 75 min, resulting in the formation of CNCs with average yield percentages ranging from 38.1% to 69.9%. Separation of the acid from the CNC was facilitated by a centrifugation process followed by dialysis against deionised water. Uniform dispersion was then achieved using ultrasonication. A variety of analytical techniques were employed to investigate the morphological, chemical, thermal, and physical properties of the isolated CNCs. Among these techniques, attenuated total reflection-Fourier-transform infrared spectroscopy (ATR-FTIR), energy-filtered transmission electron microscopy (EF-TEM), thermogravimetric analysis (TGA), and X-ray diffraction (XRD) were utilised to analyse the CNCs. The findings indicated that the separated CNCs exhibited a rod-shaped morphology, measuring between 78 and 358 nm in length and 5 and 16 nm in diameter, and also exhibited high crystallinity (75–89%) and good thermal stability. The extracted CNCs were mixed with polyvinyl alcohol (PVA) and glycerol to assess their reinforcing effect on plastic films. The prepared composite film exhibited improved mechanical properties and thermal stability. Incorporating CNCs led to a 31.9% increase in the tensile strength and a 42.33% rise in the modulus of elasticity. The results from this research proved that CNCs can be extracted from postconsumer mixed fabrics as a potential solution to effectively address the mounting concerns surrounding waste management in the textile industry and also provide avenues for enhancing the qualities of eco-friendly composite films.

## 1. Introduction

The transformation of industrial and postconsumer waste into valuable products is a continuously expanding field of research. Numerous initiatives are being implemented globally to promote the idea of converting waste into wealth. The textile industry’s fast-paced fashion cycle has significantly contributed to the excessive levels of material consumption and waste generation. According to various sources, it has been approximated that the United States discards around 32 kg of clothing per person annually. Shockingly, approximately 85% of this clothing ends up in landfills [1,2]. The reason is that most clothing exists in blends of cotton and polyester or more fibres that are hard to fractionate and/or separate from each other and sort according to their type, which hinders their recyclability and reintroduction to the supply chain [3]. 

Consequently, there has been a growing research focus on the recycling and reutilisation of blended polyester–cotton fabric in recent times. Generally, there exist two primary approaches for achieving the recycling of polyester–cotton fabric: one involves direct recovery without separating polyester and cotton, as proposed by Zou et al. [4], while the other method involves recovering polyester or cotton individually based on their distinct chemical properties, including dissolution [5,6,7], hydrothermal separation [8,9,10], hydrolysis [11,12], and enzymolysis [13,14]. The former yields textiles of lesser quality, including items like carpeting, padding, and nonwoven goods [15], as the mechanical treatment easily damages the strength of the fibres. The latter usually involves depolymerising one component in the mixture whilst recycling the other. Utilising cotton–polyester waste for the extraction of cellulose nanocrystals is a viable option, especially considering the high cellulose content present in cotton. Through scouring and bleaching processes employed in the production of clothing items, the cellulose content can be elevated up to 99% [16]. Efficient separation of blended textiles can be achieved through acid treatment and mechanical forces due to the contrasting acid resistance properties of cellulose and synthetic fibres [17]. Cellulose is easily degraded by strong acids, whilst synthetic fibres such as polyester resist acid attacks. Consequently, by subjecting the blended textiles to acid hydrolysis and the use of ultrasonication, it becomes feasible to upscale blended postconsumer textile waste into cellulosic nanocrystals and recover synthetic fibres. In addition, the recovered polyester can be recycled into new synthetic fibres through the process of melt spinning, promoting the circular economy of textiles [18].

CNCs are a prevalent natural biological polysaccharide that hold great promise in numerous applications due to a variety of key features, such as exceptional strength, high modulus, extensive surface area, unique optical properties, environmental friendliness, and biodegradability [19]. Research has demonstrated the isolation of CNCs from cotton in textile waste materials and industrial by-products [1,17,20,21,22,23,24,25]. However, the efficacy of this procedure has not been evaluated on different structures of postconsumer blended fabrics, which more accurately represent textile waste. Postconsumer fabrics encompass various chemicals that are not disclosed on the available labels and have undergone diverse chemical and mechanical pressures throughout their lifespan. The impact of these factors, in conjunction with the fabric structure, on the composition and characteristics of isolated CNCs remains uncertain. Additionally, discarded textiles primarily consist of a combination of different mixtures of fibres, and the degradation extent differs from different fabric structures. While research is currently being conducted on methods to efficiently recover fibres individually from blended textile waste, it remains uncertain if they can be utilised for extracting CNCs from cotton without compromising or damaging the synthetic component. While other researchers have successfully extracted coloured CNCs from dyed textile waste [16], the dye in the fabrics is still perceived as a disadvantage, as it remains in the final product and is known to have some influence on the properties of the CNCs, particularly the colloidal stability of the CNC suspension [26]. Bleaching is therefore essential to guarantee the quality and purity of the CNC, as it significantly impacts both its economic significance and implementation. A thorough examination of their characteristics is necessary to evaluate the suitability of postconsumer waste textiles as potential sources for cellulose nanocrystal isolation. The extraction of CNCs from postconsumer textile waste allows resources to be reused and repurposed to minimise the environmental impact and promote economic sustainability. The transparency, high elastic moduli, aspect ratios, and significant surface areas of CNCs improve their effectiveness as reinforcement additives in polymers like PVA [27]. PVA has found extensive utilisation across various fields, including drug delivery, adhesive production, packaging, absorption of heavy metal ions, and reduction of acoustic noise. However, its poor mechanical and thermal properties limit its use. PVA enhanced with cellulose fillers typically exhibits enhanced mechanical, thermal, and barrier characteristics in comparison to the pure polymer [28,29,30]. PVA is known for its biodegradability, and when reinforced with CNCs, the resulting composites maintain this eco-friendly characteristic. The incorporation of CNCs enhances the biodegradability of the composite materials. By using CNCs as reinforcing agents in PVA composites, manufacturers can improve resource efficiency, as CNCs offer high aspect ratios and excellent mechanical properties, allowing for the production of strong and durable materials with a reduced overall material consumption. 

Previous researchers have extracted CNCs from textile waste; however, no work on the effect of the fabric structure or mixed shoddy waste on the extraction and physicochemical properties of CNCs has been reported. The present study extracted CNCs from various postconsumer fabrics containing a blend of polyester and cotton fibres and were in a woven, knitted, or mixed shoddy state. Fabrics were pretreated with a two-stage dye stripping process that targeted the removal of dye and any other impurities present in the fabrics. The hydrolysis of fabrics using sulphuric acid resulted in the concomitant retrieval of the synthetic portion. Following this, an evaluation of the physical and chemical attributes of the isolated CNCs was conducted, with a comparison made to previously reported information. The influence of the fabric structure on the yield and overall crystallinity of the CNCs was also investigated. Additionally, an analysis was conducted on the recycled polyester obtained from the textiles to assess its suitability for use in the production of new polyester fibres. Subsequently, the isolated cellulose nanocrystals (CNCs) were utilised in enhancing the characteristics of polyvinyl alcohol (PVC) films.

## 2. Materials and Methods

### 2.1. Material

The postconsumer waste fabrics were collected from a local landfill in Durban, South Africa. Three distinct materials were employed in this study, namely a yellow-dyed knitted, as well as a blue-dyed woven fabric, and a shoddy of mixed-coloured fabric, all comprising polyester and cotton. The fibres’ composition was verified through Fourier-transform infrared (FTIR) analysis, as detailed in the Supporting Information. The FTIR spectrum displayed the unique peaks and bands associated with both fibre components. The distinctive peaks of cotton are observed at a wavenumber of 3340 cm^−1^, which is designated as the hydroxyl group (O–H) stretching in cellulose, and at 2898 and 1336 cm^−1^, which are associated with the C–H symmetric stretching and C–H bending in methylene (–CH_2_^−^) and methyl (–CH_3_) groups present in cellulose, respectively. The C–O stretching vibration of the β-1,4-glycosidic linkages (C–O–C) in cellulose is represented by the peaks observed at 1106 and 1020 cm^−1^. The bands for polyester are also clear on the spectra. Aliphatic C–H stretching bands are observed around 2899 cm^−1^. The intense peak at 1715 cm^−1^ in polyester is attributed to the stretching vibration of the (C=O) carbonyl group in the ester linkage. Additionally, the stretching of the aromatic ring C=C is detected at 1503 cm^−1^. The C–H bending in polyester is assigned to peaks at 1405 cm^−1^. Furthermore, the stretching vibration of the ester linkage (C–O–C) in polyester is observed at 1249 cm^−1^, while the C–H bending in the aromatic ring and the C–H bending in the C–CH_2_^−^ fragment are identified at 871 and 718 cm^−1^, respectively. The determination of the fibre content in the mixed fabrics and shoddy was conducted by selectively degrading the cotton fibres using 75% sulphuric acid, following the guidelines outlined in ASTM D629-08 [31] (refer to the Supporting Information). Sodium dithionite (≥82% RT), H_2_SO_4_ (ACS reagent, 95.0–98.0 wt%), sodium hydroxide pellets (ACS reagent, ≥97.0%), hydrogen peroxide solution (30% *w*/*w* in H_2_O with a stabiliser), glycerol, and PVA (Mw 89,000–98,000, 99% hydrolysed) were all acquired from Sigma Aldrich (Johannesburg, South Africa) and used in their arrived state without further purification. MERPOL HCS surfactant was used in each experiment as a wetting agent and was acquired from Sigma Aldrich (Johannesburg, South Africa).

### 2.2. Discolouration 

A two-step dye stripping process was used to remove dyes from the textile waste. The method was modified from the work reported by Li and co-authors [32]. In step 1, 20 g/L sodium dithionite was mixed with 10 g/L NaOH and 0.2 mL MERPOL surfactant in a 50 mL Erlenmeyer flask. Small pieces of waste (approx. 1 cm^2^) were added to the mixture at a material liquor ratio (MLR) of 1:30. The solution was placed in a water bath and maintained at a temperature of 110 °C for 90 min; after which, the samples were washed with hot deionised water, followed by cold water. In step 2, the fabrics were stripped in a bath containing 30 g/L hydrogen peroxide and 8 g/L NaOH, at MLR OF 1:30 at a temperature of 90 °C for 60 min. After the reaction, the samples were washed in hot deionised water, followed by a cold wash. The samples were left to dry for 24 h in a fume hood.

### 2.3. CNC Preparation

**Figure 1:**
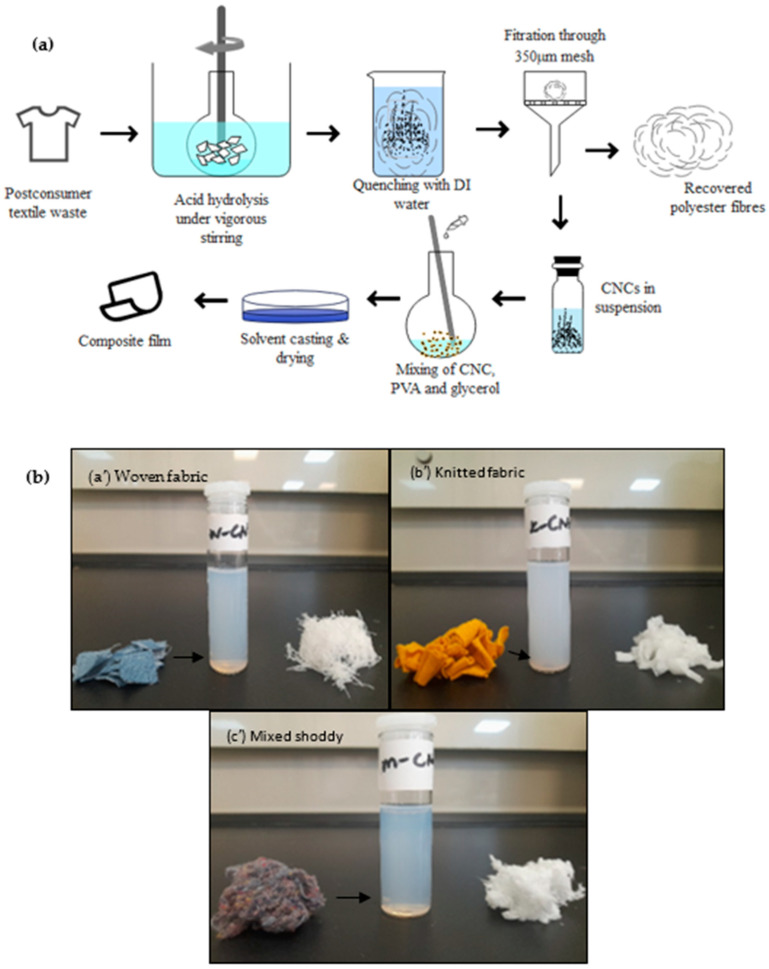
(**a**) Schematic illustration of the isolation and separation process of CNCs from the polyester/cotton postconsumer waste. (**b**) Pictures of polyester/cotton fabrics at the start, and the products after acid hydrolysis and filtration.

Cellulose nanocrystals were isolated from waste fabric cotton fibres by eliminating the amorphous sections using concentrated sulphuric acid. Acid hydrolysis was conducted in a round-bottom flask immersed in an oil bath with 64% H_2_SO_4_ at 50 °C. The material-to-liquor ratio was 1:10 (g/mL). A flow diagram of the procedure is illustrated in Figure 1a. An overhead mechanical stirrer was employed to stir the reaction mixture at a speed of 500 rpm. After an elapsed time of 60 min, the reaction was brought to an end by diluting it with 10-fold ice-deionised water. The mixture was stirred with a glass rod for a minute and then filtered through a 350 µm metal mesh under vacuum to separate the polyester fibres from the CNC suspension with acid excess. The polyester fibres underwent a thorough washing process using deionised water, and the initial washings were collected and combined with a solution to retrieve a portion of the hydrolysed material that had adhered to the polyester fibres. The recovered polyester fibres were then air-dried for 24 h and waited for further analysis. The suspension underwent centrifugation at a speed of 9000 rpm for 15 min. Subsequently, it was subjected to three rinse cycles with deionised water to eliminate the majority of the surplus acid until the supernatant turned turbid. The resultant precipitate of CNCs and residual acid was dispersed in deionised water to make 200 mL and stirred with a magnetic stirrer for 5 min. The CNC solution was then transferred to dialysis membranes (avg flat width 35 mm, MWCO 12,000 Da) and underwent dialysis with deionised water until the pH was between 6 and 7. The deionised water was changed every 12 h. An ultrasonic homogeniser (Q500, Qsonica, Newtown, CT, USA) operating at 500 W and 20 kHz was employed to sonicate the suspension in an ice bath at 60% amplitude for 5 min. The final suspension of CNCs was stored under 5 °C for further analysis. For analysis, a small proportion of the CNCs was freeze-dried. 

### 2.4. Yield Calculation

Triplicate measurements were conducted to determine the yield of the CNC suspensions in each sample, following the calculations reported by Maciel et al. [24]. Prior to drying, 20 mL of each suspension was carefully transferred into plastic vials that had been dried and weighed beforehand. The vials were frozen for 24 h at −50 °C and then freeze-dried. After this, the vials with freeze-dried fibres were weighed, and the yield was given according to Equation (1):

(1) $$CNC\;Yield\%=\frac{\left(M_{2}-M_{3}\right)\;*\;V_{1}}{M_{1}\;*\;V_{2}}\;*\;100$$

where *M*_1_ is the mass of initial waste used, *M*_2_ is the total mass of freeze-dried CNC plus vial, *M*_3_ is the mass of the empty vial, *V*_1_ is the volume of prepared CNC suspension, and *V*_2_ is the volume of sample CNC used in the vials.

### 2.5. Composite Film Preparation

PVA/CNC films were fabricated using the solvent casting technique. A solution was prepared by dissolving 3 g of polyvinyl alcohol (PVA) in 100 mL of DI water under constant overhead stirring (500 rpm) at a temperature of 100 °C for 1 h. To the above mixture, 1 mL of glycerol, a plasticising agent, was added under continuous stirring until complete gelatinisation of the mixture was achieved. Then, different weights of CNCs (0.03 g, 0.09 g, and 0.15 g) were added to the PVA solutions to make PVA/CNC 1, 3, and 5 (part per hundred with respect to dry PVA), respectively. A homogeneous solution was obtained by stirring the solution with a magnetic stirrer for 1 h. Subsequently, the solutions were poured onto a glass petri dish measuring 120 mm in diameter and left to dry at room temperature (24 ± 2 °C) for 72 h. The resultant composite films were labelled as neat PVA, PVA/CNC1, PVA/CNC3, and PVA/CNC5.

### 2.6. Material Characterisations

#### 2.6.1. Scanning Electron Microscopy

Observation of the surface morphology of the recovered polyester and the PVC/CNC films was conducted using a scanning electron microscope Phenom Pharos (Thermofisher scientific, Waltham, MA, USA) at a voltage of 15 kV. Before the SEM observation, the specimens were mounted onto a metal stub with the use of carbon tape and subsequently covered with a thin gold coating using a Q150RES sputter coater (Quorum, East Sussex, UK).

#### 2.6.2. Transmission Electron Microscopy

Morphology of the suspended CNC was done with a Transmission Electron Microscope (JEOL JEM 1400 Peabody, MA, USA), and imaging was done at 120 kV in the bright field mode. The suspended CNC sample was first diluted to 0.1% consistency. A nickel formvar-coated grid was positioned on top of a drop of sample for 5 min (this was done on a piece of parafilm in a petri dish). Thereafter, the grid was removed, and the excess sample was wicked away with a piece of filter paper. The grid was then floated on top of a drop of 2% UA, with the sample side facing down, for 30 s. The grid was then removed, the surplus stain was removed, and the grid was left to air dry for at least 10 min, with the sample side facing up. During staining, dark conditions were simulated (covered the sample with a box to block out the light) to prevent the stain from precipitating in the sample.

#### 2.6.3. Hydrodynamic Properties

The hydrodynamic properties of the CNC suspensions were obtained using a Malvern Zetasizer Nano ZS (Malvern, UK). A refractive index of 1.469 and an absorption value of 0.001 were used. A 2.00 mL aliquot of 0.1 wt% consistency for each of the stock solutions was filtered through a 0.45 µm syringe filter. Each filtrate sample was then analysed for its particle size and surface charge properties. The hydrodynamic properties are provided as the mean ± standard deviation (SD, *n* = 3).

#### 2.6.4. X-ray Diffraction

The diffractograms of freeze-dried CNCs were measured using an X-ray diffractometer (Rigaku MiniFlex 600, Akishima-shi, Tokyo, Japan) with a Cu Kα X-ray generator source (λ = 0.154 nm) at 40 kV and 15 mA. Continuous scanning was conducted within the range of 10 to 60° 2θ, with a step size of 0.02° and a scan speed of 10° 2θ/min. The degree of crystallinity was calculated using the empirical XRD peak deconvolution method suggested by Hermans et al. [33] and cited by Gusev et al. [34] and Salem et al. [35]. This method was chosen as a more accurate way to calculate the CNC crystallinity, as encouraged by French and Terinte et al. [36,37]. Peak separations were conducted through Gaussian deconvolution utilising OriginPro 2024 graphics and analysis software. Linear backgrounds were subtracted from the curve fitting process, which was determined by convoluting the sharp peaks. The amorphous component was characterised using the broad Gaussian peak approach. The crystallinity index was determined by evaluating the proportion of the combined area of crystalline peaks to the total area [19] using Equation (2):

(2) $$CI=\frac{A_{cr}}{A_{sample}}$$

where *CI* is the crystallinity index, *A_cr_* is the integral sum of the intensities of the crystalline peak areas, and *A_sample_* is the total area of the sample.

The mean particle size of the cellulose nanocrystallites was determined based on the X-ray diffraction data using Scherrer’s Equation (3) [38], where the average crystallite size, L, is given by

(3) $$L=\frac{K\lambda }{\beta \;Cos\;\theta }$$

where *λ* stands for the X-ray wavelength (0.154 nm), *β* represents the peak width of the diffraction peak profile at half-maximum height due to the small crystallite size in radians, and *K* is a constant linked to the crystallite shape, conventionally set at 0.9.

#### 2.6.5. Fourier-Transform Infrared Analysis

Infrared spectra of the fabric materials, the shredded shoddy, and recovered PET were measured using a FTIR spectrophotometer (Perkin Elmer, Shelton, CT, USA) with a universal attenuated total reflectance (ATR) accessory. Spectral measurements were taken across the 4000–600 cm^−1^ range, with a resolution set at 4 cm^−1^ and an average of 4 scans.

#### 2.6.6. Thermogravimetric Analysis

Thermogravimetric analyses on the PVC/CNC films were performed with a Simultaneous Thermal Analyser (Perkin Elmer STA 600, USA). The test samples of weights between 10 and 30 mg were heated under nitrogen air at a flow rate of 20 mL/min from a temperature of 30 °C up to 600 °C at a heating rate of 10 °C/min. STA Pyris series software (version 11) was used to generate the TG and DTG data, while OriginPro 2024 software was used to generate the plots.

#### 2.6.7. Tensile Measurements

The tensile strength and Young’s modulus properties of the composite films were analysed using a Dynamic Mechanical Analyser (TA Instrument Model DMA Q800). The procedure was carried out at a ramp force of 0.5 N/min to 18 N following an ASTM standard D638-14 [39]. Specimens of the width 10 mm and 25 mm in length of the composite film were tested at room temperature using a constant crosshead speed of 50 mm/min. A total of 5 test specimens were examined for each sample, and the mean value was recorded.

## 3. Results and Discussion

### 3.1. Pretreatment of the Postconsumer Waste

**Figure 2:**
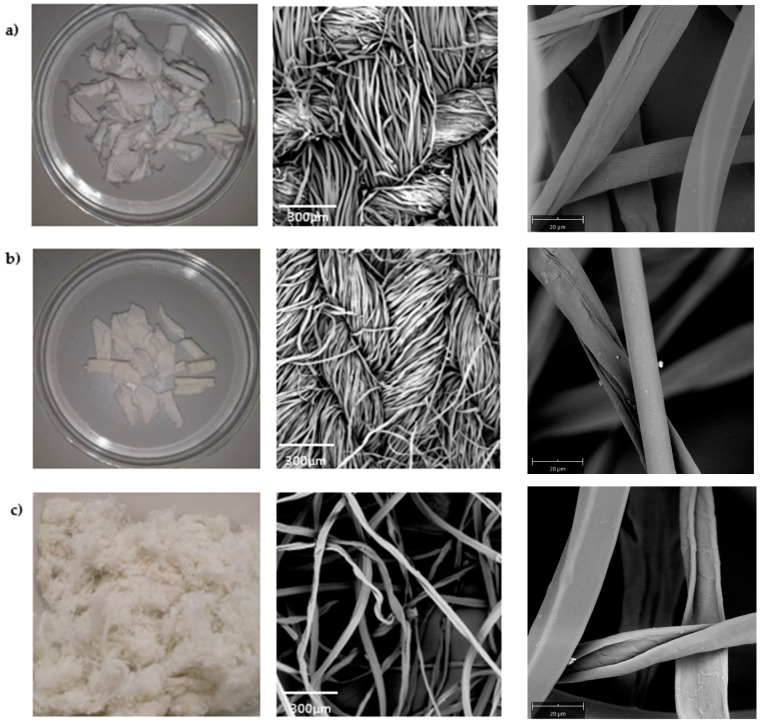
Photographs (**left**) and SEM micrographs ((**centre**) and (**right**)) of samples of (**a**) woven fabric waste, (**b**) knitted fabric waste, and (**c**) mixed waste.

Figure 2 displays the photographs (left) and SEM images (centre and right) of the materials after decolourisation. In particular, Figure 2a,b (centre) exhibit the surface of the cotton/polyester cloth waste, characterised by a uniform weave and knit pattern, respectively, and Figure 2c is a mixed shoddy of various cotton/polyester fabrics. In highlight, the SEM micrograph presents a cotton fibre, which is observed by the grooves and microfibrils and also highlights a fairly smooth and regular appearance of the polyester fibre in all samples. The pretreatment managed to remove the dyes from the samples. Stripping efficiencies of over 90% were achieved, resulting in white samples that were then used for the extraction of the CNCs. Details on the stripping efficiencies are shown in the Supplementary Information. The SEM results did not show any appearance of any damage to the fibre surfaces. 

### 3.2. Characteristics of the Extracted CNCs

**Figure 3:**
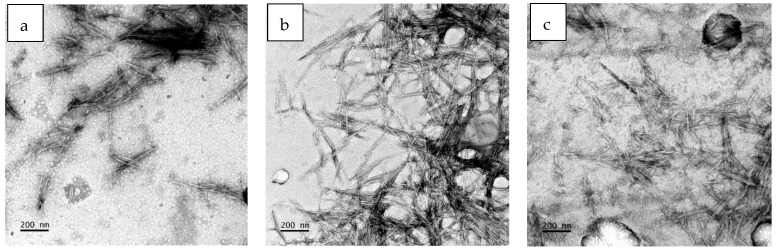
SEM images of extracted cellulose nanocrystals: (**a**) w-CNC, (**b**) k-CNC, and (**c**) m-CNC.

Sulphuric acid hydrolysis is considered the conventional and efficient technique for extracting cellulose nanocrystals (CNCs) from cotton-based textile waste [40]; thus, it was used for this work. The degradation of PET under the conditions utilised in this study is negligible, despite the potential for hydrolysis when PET is exposed to sulphuric acid in harsh conditions. This was proven by Ruiz-Calda and co-authors in their study where their recovered PET had a slight change in the number-average molecular weight; however, their intrinsic velocity and weight-average molecular weight remained unchanged [16]. The CNC TEM photographs are shown in Figure 3. The CNCs appeared as elongated rod-like structures with well-defined edges. The individual nanocrystals exhibited a uniform shape and size due to the controlled synthesis method that was employed in this study. The amorphous regions were affected by the sulphuric acid solution and vigorous stirring. Consequently, the hydrogen bonding connections among the cellulose molecules experienced a gradual weakening, eventually leading to their profound rupture [41]. Sulphuric acid can rapidly infiltrate the crystalline region’s surface, leading to a uniform hydrolysis process that disrupts the lengthy molecular chain of cellulose. The extent of cellulose crystal degradation varied depending on the structure of the sample. As shown in Table 1, the length of the CNCs differed with the sample. The obtained cellulose nanocrystals from k-CNC had much smaller dimensions compared to the CNCs from the woven and shoddy materials. k-CNC had a length ranging from 78 to 194 and a width ranging from 5.40 to 9.66 nm. The reduction in the size of the crystals was attributed to the uniform penetration of the sulphuric acid, which resulted in degradation of the cellulose polymer chains. The different nanocellulose dimensions can be attributed to their distinct constructions. Woven fabrics, especially on denim, are more densely compact, making the fibres less prone to unravelling compared to knitted fabrics. Knitted fabrics, on the other hand, are constructed with interlocking loops of yarn, which gives the fabric a loose structure and more air spaces in between loops. CNCs from the w-CNC were rod-like crystals with a 6–16 nm width and 135–358 nm length. The CNCs from the m-CNC sample had dimensions ranging from 165 to 295 nm in length and 12 to 16 nm in width. Irrespective of the source, the size ranges of all samples aligned with the dimensions documented in the TEM data on CNCs derived from cotton [19]. The results are consistent with the CNCs produced by Ruiz-Caldas et al., who extracted CNCs from dyed postconsumer waste [16]. 

The CNC yields for every fabric type are outlined in Table 1. The acid hydrolysis process plays a crucial role in the extraction of cellulose nanocrystals (CNCs) from blended fabrics. The 64% concentration of the sulphuric acid used could break down the cotton in the mixed waste in their fabric state. The yield from the knitted sample, k-CNC, of 53.5% was higher than the yield from the woven 44.8% or the mixed shoddy 22.4%. This was attributed to the high percentage of cotton in the knitted sample compared to the other samples. The acid-to-cellulose ratio significantly influenced the properties and morphology of the CNC produced. A more comparable analysis was to check the yield per gram of cotton present in the sample. The knitted sample had a yield of 69.5% per gram of cotton used. This was slightly above that of the woven fabric, which was 66.3%. The difference can be attributed to the different fabric structures. Woven fabrics have a more compact structure compared to knitted fabrics, thereby limiting the penetration of acid. The defining factor of weaves is the abundance of interlacements between the warp and weft yarns as compared to knitted fabrics, which have loops [42,43]. The cotton content also resulted in a higher sulphuric acid-to-cotton ratio in the fabrics with lower cotton contents. As the hydrolysis progresses, the sample with more cotton will have a less material-to-liquor ratio as the cellulose fibres are broken down. However, if the sample has more polyester fibres, which are not affected by the acid, the material-to-liquor ratio remains high, resulting in a slight change in the acid penetration to the cellulosic fibres. The yield was comparable to previous studies conducted using acid hydrolysis on textile waste cotton [21,44]. The reduced CNC yield observed in the case of mixed shoddy fibres, m-CNC, may be attributed to ineffective hydrolysis. This can be attributed to the fact that fibres have a larger volume compared to fabrics, thus necessitating a higher material-to-liquor ratio. Additionally, the fibres and yarns would entangle on the mixing rod, reducing the mixing efficiency. Thus, more fibres are likely to be left adhered on the PET surface and washed off during separation and washing. This implies that the type of fabric structure and cotton fibre content do influence the yield and length of the extracted CNCs. Thus, it is concluded that knitted fabrics with higher cotton contents should potentially be used more to effectively produce CNCs on a larger scale. Furthermore, the technique demonstrated the ability to successfully extract CNCs from textile waste in its original fabric state, resulting in significant yields. Consequently, this eliminates the need to shred the waste into fibres before recycling, streamlining the overall recycling process.

The recovered polyester was not subjected to additional analysis or processing, as this task was not within the scope of this particular study. However, from Table 1, it is shown that PET can be recovered with a yield close to 98%. Further analysis is required to assess its reuse in making new yarns.

The stability of CNCs dispersed in aqueous suspensions was evaluated using dynamic light scattering (DLS). The CNCs had a hydrodynamic size ranging from 153 to 158 nm. Although DLS measures an apparent hydrodynamic size, it should be noted that this size does not accurately represent a physical dimension; however, a good hydrodynamic diameter for cellulose nanocrystals is typically in the range of 100 to 300 nanometres [45]. Our reported sizes were faintly higher compared to the hydrodynamic sizes reported by Ruiz-Caldas and co-authors [16]; however, they were in the range reported by Wang and co-authors [46].

Table 1 displays that CNCs derived from different textile waste structures exhibited a comparable average zeta potential of approximately −32 mV. The high zeta potential indicates that the suspensions have high dispersion and colloidal stability. The structure of the textile materials did not have much difference in the zeta potential and surface charge of the recovered CNCs. This is probably because the surface and the ultrasonication conditions used were the same and managed to disperse the crystals more evenly in the suspension. This charge was fairly high compared to the results obtained by Huang and co-authors [47] but quite similar to those reported by Csiszár and Sebestyén from bleached cotton [48]. The magnitude of the electrostatic repulsion between crystals is responsible for this occurrence. The negative charge can be attributed to the sulphate groups originating from the acid, and these are responsible for the electrostatic repulsion between crystals, preventing aggregation and promoting stability within the suspension.

**Figure 4:**
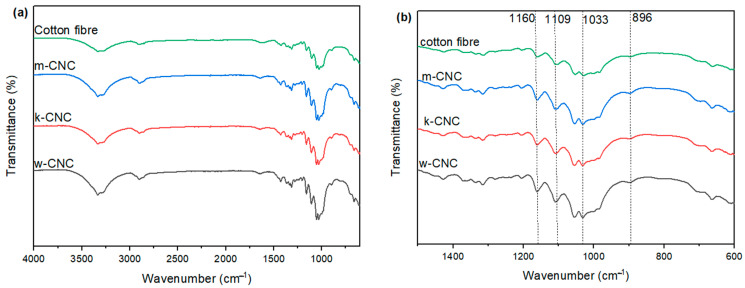

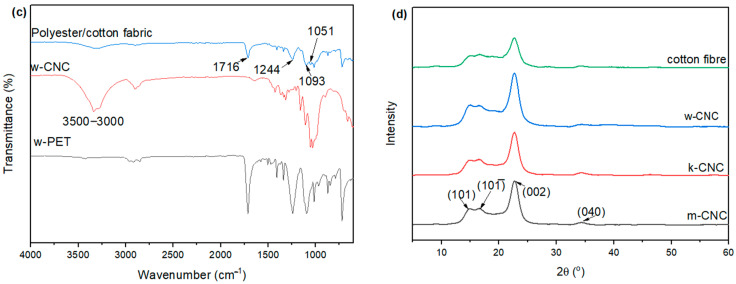
(**a**) FTIR spectra of waste cotton fibre and the extracted CNCs, (**b**) zoomed image of the highlighted area of concern, (**c**) spectra of woven polyester/cotton fabric (w-CNC) and polyester fibre recovered from the woven fabric, and (**d**) XRD diffraction patterns of the extracted CNCs and waste cotton fibre.

The CNCs produced exhibit a high level of purity. In Section 3.4 (Figure 4a), a detailed explanation will be given about how the CNCs exhibit a similar pattern to the fingerprint of pure cellulose, without any other peaks that could be attributed to impurities. This finding indicates that the processes of decolourisation, isolation, and dialysis purification employed in this study were highly efficient in producing cellulose nanocrystals with minimal impurities or contaminants. Moreover, the high degree of crystallinity typically associated with a purer form of CNCs serves as further confirmation of the effectiveness of the purification methods. The ultimate CNC suspensions possessed a neutral pH of 6.95 (w-CNC), 6.83 (k-CNC), and 7.10 (m-CNC), signifying their purity and safety for operational use.

### 3.3. XRD Patterns of CNC from Bleached Postconsumer Waste

Figure 4d shows the XRD patterns of the obtained w-CNC, k-CNC, m-CNC, and waste cotton fibres. Four characteristic diffraction peaks were observed at 2θ = 14.7°, 16.6°, 22.8°, and 34.4°, corresponding to the crystal lattice planes (110), (1–10), (200), and (040) [16,49]. These traits are commonly associated with cellulosic materials that are rich in the Iβ crystalline allomorph, which is a dominant crystalline form of cotton fibre [50]. Comparatively, the (200) plane of CNCs exhibited a more distinct 22.6° peak, signifying an enhanced level of perfection in the crystal lattice of the original cellulose in contrast to cotton. The intensity of the peak corresponding to the (110) plane (2ϴ = 14.7°) increased significantly, and it was observed to be distinct from the (1–10) plane (2ϴ = 16.4°) in the extracted CNCs. The proximity and comparable intensity of the first two diffraction peaks indicate their correlation. Compared to the waste cotton fibre, the crystallinity of w-CNC, k-CNC, and m-CNC increased from 70.3% to 86.1%, 89.9%, and 74.5%, respectively (Table 2). The treatment period with sulphuric acid caused extensive damage to the amorphous region, ultimately resulting in a higher level of crystallinity. The crystallinity of the knitted fabric was higher than that of the other samples. This can be attributed to the structure of the knitted material, which allowed the free circulation of acid to break down the amorphous region, increasing the crystallinity as a result. The crystallinity of m-CNC only increased to 74.5% from 70.3% of waste cotton fibre. The slight increase observed can be linked to the damage caused by sulphuric acid. Due to the low cotton content in the sample (Table S1, Supplementary Information), the cotton-to-acid ratio was lower compared to the other samples. This allowed more acid to access the cellulose molecules at a faster rate. Furthermore, the high surface area of this particular sample may have contributed to this phenomenon. Consequently, the surplus acid led to the destruction of the amorphous regions and peeling off of the crystalline regions, resulting in a significant weight loss. As a result, there was a decrease in the crystallinity over time and the overall yield for m-CNC. The crystallinity obtained for all the samples was higher than the one obtained by Haouaoche and co-authors [51]. However, the crystallinity was in range with the work reported by Yue and co-authors [50].

Table 2 provides a summary of the estimated crystallinity index, crystallite cross-sectional dimensions calculated using the Scherrer equation, and the corresponding d-spacings. In general, the size of the crystallite remained constant, with the primary factor influencing size being the source material. The crystallite sizes obtained from knitted fabric were 3.81 nm, whilst those from woven fabric and shoddy were 3.52 and 3.85 nm, respectively. The calculated crystallite size did not exhibit significant variations, yet the TEM images provided a more intricate measurement of the crystallite size. The dimensions of the crystalline regions were in accordance with values that have been previously published [16,50,52]. The (200) and (1–10) diffraction peaks exhibit associated d-spacings and relative intensities that are associated with the arrangement of β-1,4-linked glucan chains that form the crystalline lattice of CNCs. This indicates a level of neat alignment and order that results in good mechanical properties in CNCs.

### 3.4. FTIR of CNC Extracted from the Postconsumer Waste

The FTIR spectra were employed for the examination of the surface functional groups existing in both CNCs and waste cotton fibres. Figure 4a,b compare the FTIR spectra of postconsumer waste cotton and the CNCs extracted from the different structures of the samples. The spectra observed in the CNC samples are practically identical, owing to the effectiveness of the extraction process and the subsequent transformation into cellulose nanocrystals. However, upon comparing different CNC samples to the waste cotton, slight disparities were detected in the FTIR spectra. Stretching vibrations can be observed in the hydroxyl (–OH) groups, as well as the methyl and methylene (C–H) groups, which are assigned to the adsorption peaks observed between 3300 and 3600 cm^−1^ and 2900 cm^−1^ in all spectra. The band observed at 1638 cm^−1^ corresponds to the hydroxyl bending vibration resulting from the adsorption of water by cellulose molecules. The extracted CNC samples exhibited characteristic peaks associated with C–O stretching at 1160 and 1109 cm^−1^ and C–H bends at 1311 cm^−1^. The C–OH stretching band intensities were increased in the CNC samples as compared to the cotton waste (Figure 4b). A distinctive heightened band at 896 cm^−1^ is assigned to cellulose–O-S_3_Na in sulphated cellulose, whereas the band at 1033 cm^−1^ (S=O stretches) is concealed by the band of cellulosic material. This is likely a result of the incorporation of sulphate groups and the refinement of the cellulose crystal structure [26,53]. The particular signals are typically identified in sulphate CNCs with around 100–200 sulphate groups per anhydroglucose unit [53,54,55]. These results confirm the presence of the sulphate group, which was also a result of the negative surface charge of the CNCs. The structure of the starting material did not affect the resulting functional groups of the CNCs.

Figure 4c shows the FTIR spectra of the mixed polyester cotton fabric and the separated CNCs and polyester fibre. Characteristics peaks of the functional groups relating to polyester and cotton are highlighted. The FTIR data exhibit a distinct O–H stretching band from 3300 to 2900 cm^−1^, which undergoes sharpening at 3332 cm^−1^, and several peaks in the region 1200–1030 cm^−1^ indicative of the C–O–C stretching vibration associated with the glucopyranose ring. The FTIR spectrum of polyester fibres exhibits a prominent peak associated with the carbonyl group at 1716 cm^−1^, along with two additional peaks at 1244 cm^−1^ and 1093 cm^−1^, which can be assigned to the stretching vibrations of the C–O ester group. As observed by FTIR, no bands associated with polyester fibre were observed in the polyester portion and vice versa. This confirms the highly selective property of sulphuric acid towards cotton fibres, which resulted in successful separation.

### 3.5. Thermal Properties of CNCs from Bleached Postconsumer Waste

**Figure 5:**
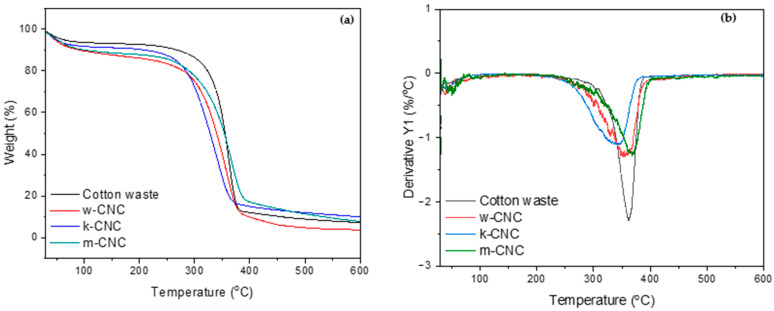
(**a**) TGA and (**b**) DTG curves for cotton and the extracted CNCs.

It is crucial to comprehend the thermal properties of cellulose to distinguish the variations in processing temperature for practical utilisation. The TGA and DTG curves of the extracted CNCs are displayed in Figure 5a,b, respectively, and their thermal stability data obtained from the DTG curves are listed in Table 3. The cellulose nanocrystal samples exhibited main degradation temperatures ranging from 344 to 366 °C, indicative of the breakdown of the primary cellulose chains.

The thermal degradation profiles of all three CNC types display almost indistinguishable characteristics showing a two-step decomposition process. The weight of all CNCs experienced a decrease of less than 5% below 100 °C, signifying the elimination of moisture through evaporation. The major degradation of the CNCs is marked by a notable loss phase that initiates at a temperature of about 233 °C (Table 3), culminating in complete degradation at approximately 400 °C for w-CNC and m-CNC and 381 °C for k-CNC. The maximum degradation temperatures, which represented temperatures at the maximum degradation rate, are shown in Table 3 and were obtained from the peak point in the DTG curves (Figure 5b). This is attributed to cellulose, which undergoes a series of reactions that involve the breaking of glycosidic bonds between glucose units at temperatures higher than 200 °C. All CNCs exhibited slightly lower thermal stability compared to the waste cotton and showed a slight decomposition shoulder at around 270 °C, which was attributed to the decomposition of the sulphate half-ester groups. This is evident from the onset degradation temperatures of the CNCs, which are slightly lower than the onset degradation temperature of waste cotton of 249 °C. The onset degradation temperature for k-CNC was the lowest owing to the high crystallinity of the sample compared to all the other samples. Consecutively, the offset degradation temperature for this sample was lower than the rest, showing that, by the temperature of 381 °C, all the cellulose had been degraded. As for w-CNC and m-CNC, their offset temperature was about 400 °C, showing that they required more heat to completely degrade. Their low stability was also evident from the lower maximum degradation temperature, which was 344 °C for k-CNC and 353 °C for w-CNC. However, the mixed shoddy showed a maximum temperature of 366 °C, which was almost similar to waste cotton fibre. This can be attributed to the almost similar crystalline properties of the sample. It could be that the acid hydrolysis was not effective in changing the thermal properties, since it still had a lot of amorphous regions similar to the cotton fibre. Earlier investigations have pointed out that the lower thermal stability of CNCs is linked to their notable specific surface area, causing an increase in free chain ends. Consequently, this accelerates the degradation process at lower temperatures [56,57]. In addition, it emerges from catalytic dehydration triggered by the acidic surface generated by the sulphate half-ester groups. By substituting the hydroxyl (OH) group with sulphate groups, the activation energy for the degradation of cellulose nanocrystals (CNCs) is effectively reduced. Consequently, the samples exhibit decreased resistance to pyrolysis [24]. The zeta potential analyses provided additional evidence to support the results, as they indicated a negatively charged surface. This observation suggests the existence of sulphate groups in the cellulosic chains. Less than 10% of char residue was observed post-exposure to a temperature of 600 °C, which eliminated the possibility of the presence of impurities, such as lignin, hemicellulose, or any other impurities that would normally elevate the char yield [58].

### 3.6. Mechanical Properties of PVA/CNC Composite Films 

**Figure 6:**
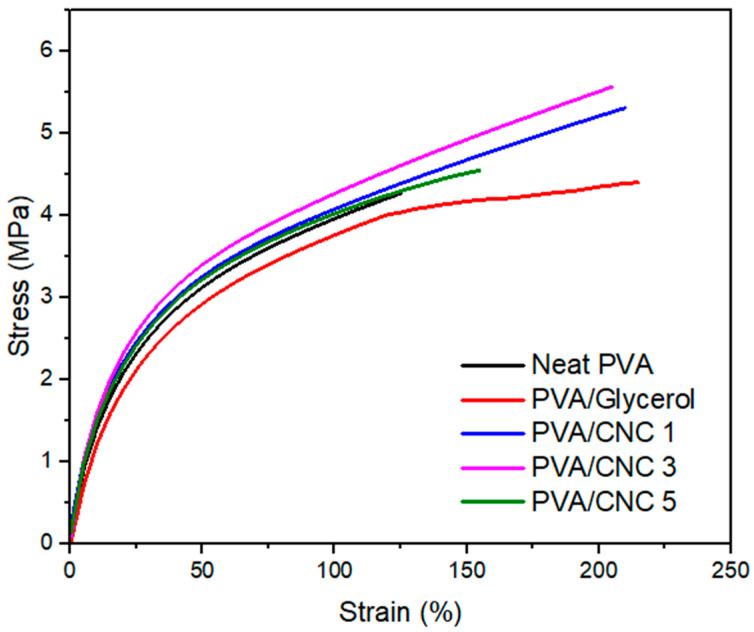
Mechanical properties of neat PVA, PVA with glycerol, and composite films.

PVA/CNC composite films were fabricated using the k-CNC, which gave better crystallinity compared to the other two samples. The mechanical properties of the films are summarised in Table 4, and their stress–strain curves are shown in Figure 6. The addition of CNC and glycerol to the PVA polymer resulted in an enhancement of both the tensile strength and modulus of elasticity. Initially, there was a 26.71% increase in tensile strength at a concentration of 1% of CNC. A peak tensile strength of 5.58 MPa was achieved at a CNC concentration of 3%, representing a significant 31.91% enhancement compared to the pure PVA film. The enhancement in mechanical characteristics can be credited to the incorporation of highly crystalline cellulose nanocrystals. Furthermore, glycerol utilised as a plasticiser implied a strong hydrogen bonding interaction between cellulose and the PVA polymer [48]. The effective interaction facilitated the proficient transmission of impressed stress onto composite films, consequently leading to an equitable dispersion of the applied stress and subsequently diminishing the magnitude of the stress intensity.

The PVA/CNC 5 composite film exhibited a subsequent reduction in tensile strength from PVA/CNC 3. The reduction observed with an increased CNC addition at higher levels may have been caused by the uneven distribution of CNCs within the film. Additionally, the flexibility of the composite films experienced a notable decline as the CNC content increased, as evidenced by their elongation at the break. A rise in elongation was detected in the PVA/glycerol film relative to the neat PVA due to the plasticising influence of glycerol in the composite films. The addition of glycerol resulted in an increase in the film’s elongation from 121.66% to 215.35%. The addition of glycerol reduces the brittleness of PVA films, making them more pliable and less prone to cracking or breaking, resulting in more elongation [48]. The increase in CNC concentration caused an increase in material stiffness, resulting in a decrease in elongation. Previous research has indicated that the heightened rigidity of the composite film restricts the mobility of PVA/CNC molecular chains, consequently decreasing the elongation at the point of fracture [26]. The films preserved excellent flexibility at a 3% concentration, enabling their potential application in food packaging. The Young’s modulus exhibited a positive correlation with the rise in tensile strength. However, a slight decline in the Young’s modulus was noted for the PVA/glycerol film. This was expected, as the glycerol reduced the stiffness of the film compared to the neat PVA film. At a CNC concentration of 3%, the modulus reached its peak at 29.69 MPa, representing a significant 42.33% enhancement over the neat PVA. The enhancement in the Young’s modulus resulted in enhanced mechanical efficiency and enhanced ability to withstand deformation when subjected to loads. The integration of CNC into the mixture not only decreased plastic deformation but also enhanced stress transmission within the composite film. Zhong et al. [26] also observed comparable patterns, where the inclusion of 5% CNC derived from indigo-dyed cotton waste resulted in a significant enhancement of the tensile characteristics of the PVA film. Our findings on tensile strength and elongation exhibited superior outcomes compared to the data presented by Srichola et al. [52]. In their study, the tensile strength was recorded at 2.37 MPa with a 5% nanocellulose loading.

### 3.7. Morphological Properties of PVA/CNC Composite Films

**Figure 7:**
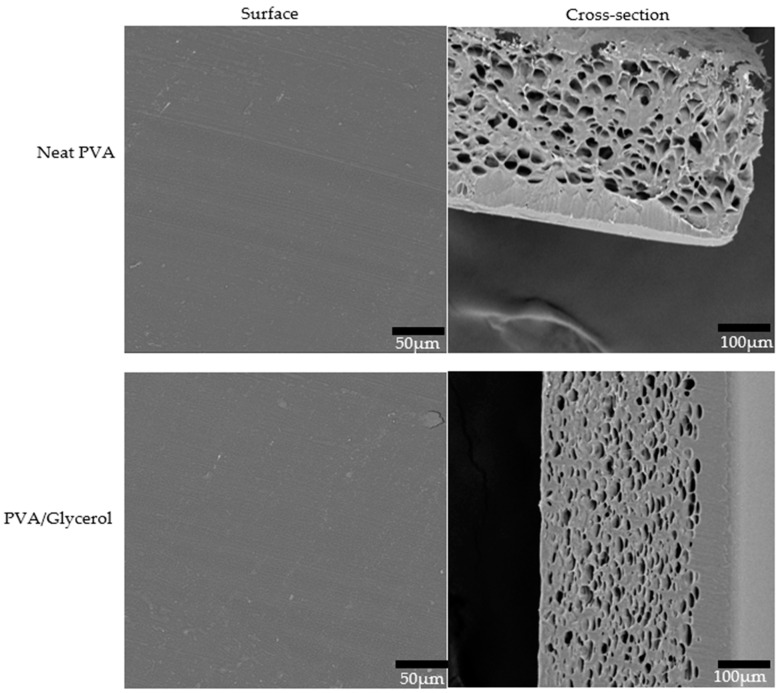

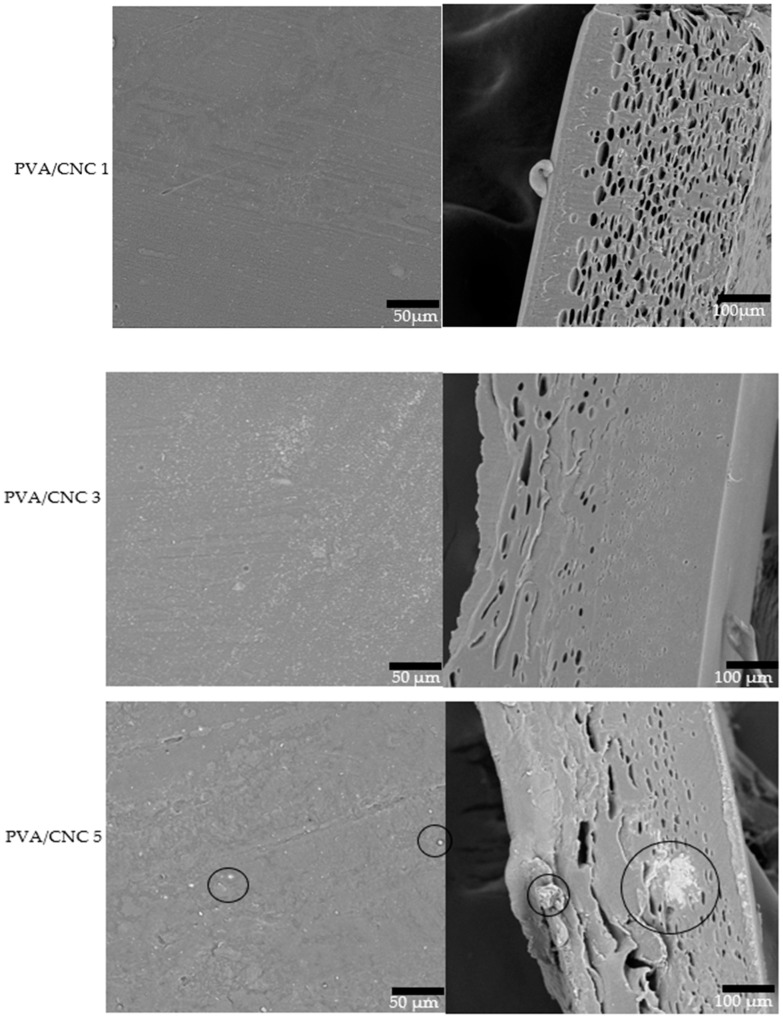
Morphological appearance of the PVA/CNC composite film: surface appearance (**left**) and cross-sectional view (**right**). Circled areas represent agglomerates.

The obtained composite films were flat, smooth, and colourless. Figure 7 displays the results of the SEM analysis conducted to examine the surface and cross-sectional morphology of the composite films. The surface of the composite exhibited a progressive roughening as the loading of CNCs increased, indicating the impact of CNC particle aggregation. No indications of phase separation or fibril plucking were observed in the images, implying a significant bond between the CNCs and PVA matrix. This adhesion is likely due to the presence of hydrogen bonding and van der Waals forces. However, PVA/CNC 5 exhibited larger particles compared to the other samples. This occurrence could be a result of the agglomeration of CNC particles at a high CNC content. Consequently, this phenomenon is thought to be responsible for the decline in mechanical properties due to the absence of a uniform dispersion of the particles. The cross-sections of the films displayed a pore structure that decreased in occurrence as the CNC content increased. This particular structure is believed to be the reason for the low mechanical properties in neat PVA films. The incorporation of CNCs and glycerol resulted in a synergistic enhancement of plasticisation and void filling, leading to an improvement in the ultimate tensile strength of the films. However, PVACNC 5 exhibited a decreased tensile strength in comparison to PVACNC 3 as a result of the agglomeration also observed in the cross-section of the films. The film’s exceptional transparency and smooth surface texture are appealing to the food packaging sector. Moreover, the superior purity of CNCs derived from clothing waste, in comparison to wood nanocellulose, presents a significant advantage for utilising these CNCs in applications that require high levels of purity. Additionally, the strong adhesion between the polymer and CNCs guarantees that the composites will not compromise the contents by shedding loose particles from the surface.

### 3.8. Thermal Properties of the Fabricated PVC/CNC Composite Films

**Figure 8:**
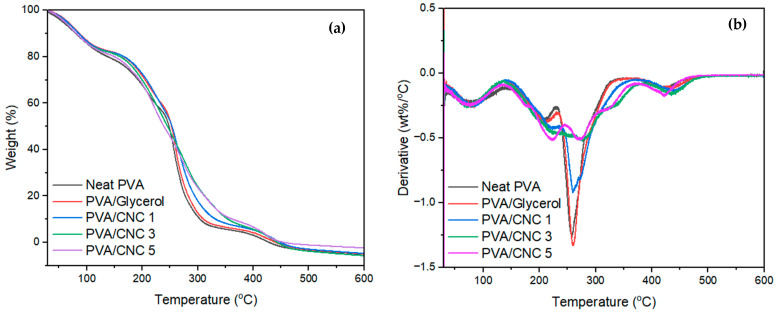
(**a**) TGA and (**b**) DTG thermographs for the PVA/CNC composite films.

The TGA and DTG curves of the composite films are represented in Figure 8. The degradation of the composite films showed four degradation stages. The first step was between temperatures 40 and 125 °C for all samples, which correlates with water evaporation (Figure 8a). The second step was between temperatures of 150 and 230 °C for neat PVA and 150 and 260 for PVA/CNC films. This is evident from the DTG curves, where the film sample exhibits a slower mass loss rate (Figure 8b). This stage was an elimination reaction that involved the elimination of side groups from the main chain and glycerol. Two distinct decomposition steps with maximum temperatures of 260 °C for PVA, 263 °C for PVA/glycerol, 270 °C for PVA/CNC 1, 290 °C for PVA/CNC 3, and 432 °C for PVA/CNC 5 were observed for the PVA degradation. The first step can be attributed to the decomposition of the side chain of PVA, while the second inflexion point corresponds to the decomposition of the main chain of PVA [59]. The initiation of structural deterioration in PVA commences within this specific area, primarily encompassing the removal of hydroxyl groups through dehydration and the creation of volatile organic compounds and conjugated, unsaturated polyenes. In the final stage, polyene remnants undergo decomposition to form alkenes, alkanes, and aromatics via intramolecular cyclisation [27,60]. It is observed that the thermal stability of the PVA was increased by the increase in the CNC content, as shown by the shift to the right of the main degradation peak at around 260 °C for the neat PVA, which was shifted to around 290 °C in the PVA/CNC 3 and 5 composite films. Glycerol is known to improve the thermal stability of some polymers in high concentrations; however, the inclusion of glycerol did not have a significant effect on the thermal stability, as shown by the almost similar TGA curves (Figure 8a). The results showed better thermal stability as compared to the work reported by Abdulla et al., as their results showed that the highest onset thermal degradation temperature was 254 °C for PVA loaded with 6 wt% CNC [30]. Similar degradation patterns were observed by Rowe and co-authors for PVA reinforced with CNC at a composition of 8% [27]. This is also in agreement with the results reported by Zhong and co-authors [26], who found that PVA composites with 10% CNC (bleached cotton) exhibited improved thermal stability. The main degradation of the cellulose from the CNC is observed clearly as a shoulder between 320 and 360 °C on the DTG curves (Figure 8b). This peak is not visible in the PVA and PVA/CNC 1 samples, as it had no CNC or the content was too low to be detected by the DTG curve.

It can be observed from the TGA curves that the incorporation of CNCs resulted in an improvement in the thermal stability of the PVA. Although 5% CNC led to a decrease in the tensile strength, it resulted in an increased thermal stability. Similar behaviours were reported by Zhong and co-authors, who reported that, although a high concentration of CNC resulted in low tensile strength, it improved the thermal stability of the composite films [26]. The shift towards higher temperatures was observed for both the onset and the maximum degradation temperatures of the composite films, showing that the CNC from postconsumer waste led to an improvement in the thermal stability of the films.

## 4. Conclusions

This investigation effectively isolated rod-like CNCs from woven fabric, knitted fabric, and mixed shoddy through chemical pretreatment: hydrolysis with sulphuric acid, followed by dialysis and ultrasonication. Prior to initiating the sulphuric acid hydrolysis process, the textile dyes and impurities from the textile waste were successfully eliminated through decolourisation treatment. The efficiency of the obtained CNC yields (38–69% cotton basis) surpassed those of the CNCs mentioned in the literature from cotton waste. The CNC properties were significantly influenced by the acid-to-cellulose ratio, as indicated by the results obtained from the samples with different cotton/polyester ratios. The structure of the sample also affected the yield of the CNC, as knitted fabrics proved to give the highest yield of 69.5% of CNCs using the discussed conditions. However, when handling the mixed shoddy, it is recommended to further break down the fibres to prevent the tangling of the yarns during mixing, consequently diminishing the efficiency of the acid hydrolysis process. The CNCs showed similar rod-like structures, ranging from 5 to 16 nm and a length of 78 to 358 nm. The cellulose I structure was evident in all samples based on the FTIR spectra. The CNCs from the knitted fabric exhibited a high crystallinity of 89.9%. The decomposition of the CNCs took place in two primary thermal decomposition phases, encompassing the heavily sulphated area (150–290 °C) and the crystalline cellulose area (260–450 °C). The thermal stability of the CNCs was slightly lower compared to the cotton fibre from the waste; however, the CNCs improved the thermal stability of the PVA when they were incorporated to make the PVA/CNC composite films. The CNCs demonstrated commendable reinforcement properties, as indicated by the notable improvement in tensile strength by 31.91% and the Young’s modulus by 42.33%. The CNC yield, reproducibility across different batches, purity level, and properties of the CNCs highlight the potential of mixed postconsumer textile waste as a viable source for CNCs. These CNCs can be effectively employed in enhancing the strength of plastic films. The present study provides evidence that this method could serve as an innovative pathway for upcycling blended fabrics, and the incorporation of cellulose nanocrystals in PVA composites positively impacts the bioeconomy by promoting sustainability, biodegradability, resource efficiency, waste utilisation, and creating market opportunities for eco-friendly materials.

## Figures and Tables

**Table 1:** Yield and hydrodynamic properties of CNCs extracted from postconsumer waste.

Sample	Hydrodynamic Size (nm)	CNC Size (nm)		Zeta Potential (mV)	Polydispersity Index (PDI)	Yield (%)		PET Recovered (wt%)
		Length	Width			g CNC per g Sample	g CNC per g Cotton	
WCNC	158.4 ± 0.85	135–358	6.30–15.96	−33.2 ± 0.55	0.277 ± 0.015	44.8	69.5	99.8
KCNC	163.1 ± 1.50	78–194	5.40–9.66	−31.5 ± 0.65	0.217 ± 0.019	53.5	66.3	98.7
MCNC	153.8 ± 2.01	165–295	12.23–16.25	−31.8 ± 1.37	0.244 ± 0.006	22.4	38.1	88.3

**Table 2:** XRD parameters of the extracted CNCs.

Sample	Crystallinity Index	d-Spacing @ Peak 002 (Å)	Average Crystallite Size (nm)
Cotton fibre	70.3	3.89	5.55
w-CNC	86.1	3.92	3.52
k-CNC	89.9	3.91	3.81
m-CNC	74.5	3.90	3.85

**Table 3:** Thermal degradation temperatures for the CNCs.

Sample	Onset Degradation Temperature (°C)	Offset Degradation Temperature (°C)	Maximum Degradation Temperature (°C)	Char Residue @ 600 °C (%)
Cotton waste	249	387	361	7.2
w-CNC	246	400	353	3.7
k-CNC	233	381	344	9.5
m-CNC	237	403	366	8.4

**Table 4:** Mechanical properties of PVA/CNC composite films at different nanocellulose concentrations (0%, 1%, 3%, and 5%).

Sample	Tensile Strength (MPa)	Elongation at Break (%)	Young Modulus (MPa)
Neat PVA	4.23 ± 0.12	121.6 ± 5.23	20.86 ± 0.34
PVA/Glycerol	4.03 ± 0.86	215.3 ± 2.56	18.96 ± 0.17
PVA/CNC 1	5.36 ± 0.63	214.6 ± 3.51	25.25 ± 0.23
PVA/CNC 3	5.58 ± 0.71	205.9 ± 6.93	29.69 ± 0.05
PVA/CNC 5	4.57 ± 0.27	157.9 ± 11.7	27.04 ± 0.15

## Data Availability

Data are contained within the article and supplementary materials.

## Supplementary Materials

The following supporting information can be downloaded at: https://www.mdpi.com/article/10.3390/polym16111495/s1: A separate document has been supplied with the Supplementary Information. Figure S1. FTIR spectra for (a) yellow knitted fabric (b) blue woven fabric, and (c) mixed shoddy. Table S1. Composition analysis of postconsumer textile waste by acid hydrolysis. Table S2. Stripping efficiency and CIE Lab coordinates of the decolorization of postconsumer waste. Figure S2 Photographs of CNC reinforced PVA composite films.
