# Supplementary material for: Physicochemical Properties of Cellulose Nanocrystals Extracted from Postconsumer Polyester/Cotton-Blended Fabrics and Their Effects on PVA Composite Films
Source: Polymers (Basel). 2024 May 24;16(11):1495. doi: 10.3390/polym16111495 (PMC11174633; doi:10.3390/polym16111495)
Supplement: Supplementary file 1 [file polymers-16-01495-s001.zip › polymers-3016152-supplementary.pdf]

SUPPLIMENTARY DATA

Physicochemical properties of cellulose nanocrystals extracted from post-consumer polyester/cotton blended fabrics and their effects on PVA composite films

Rivalani Baloyi Baloyi[1,2*], Bruce Bishop Sithole[1], Viren Chunillal[1,2]

[1]Department of Chemical Engineering, College of Agriculture, Engineering and Science. University of KwaZulu Natal, Durban, South Africa

[2]Biorefinery Industry Development facility, Council for Scientific and Industrial Research, Durban, South Africa

[*]Corresponding author, email: rivalanibe@gmail.com

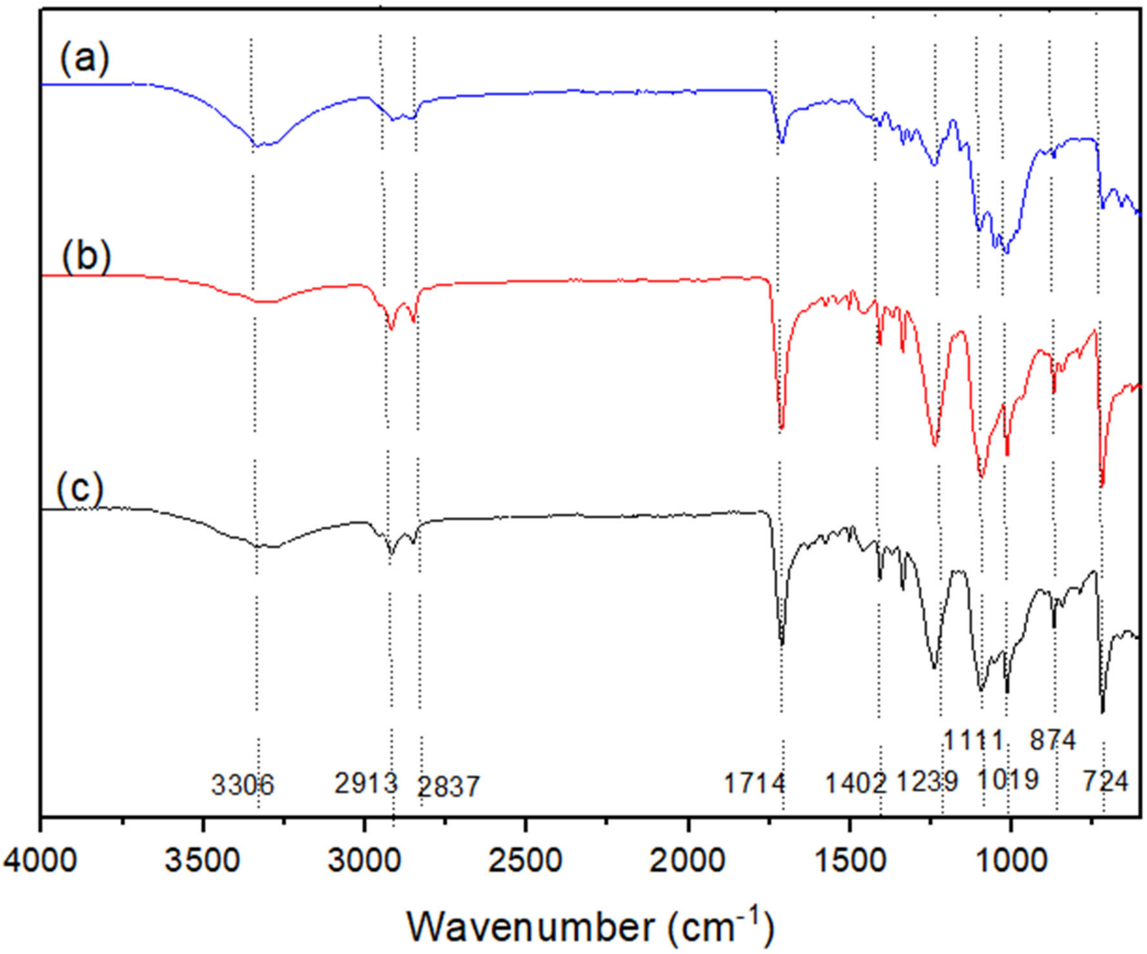

Figure S1. FTIR spectra for (a) yellow knitted fabric (b) blue woven fabric, and (c) mixed shoddy.

Table S1. Composition analysis of postconsumer textile waste by acid hydrolysis.

Sample	Fabric density (g/m^2)	Average composition of polyester (%)	Average composition of cotton (%)
**Woven fabric**	263.01	23.70	76.30
**Knitted fabric**	203.25	17.42	82.58
**Mixed shoddy**	-	39.53	60.67

Table S2. Stripping efficiency and CIE Lab coordinates of the decolorization of postconsumer waste.

Sample	CIE Lab coordinates						ΔE		Stripping efficiency (%)
	Original dyed fabrics			After dye stripping			Before stripping	After stripping	
Control	-	-	-	83.61	-0.14	0.19	-	-	-
Blue woven waste	42.27	-3.03	-14.52	79.29	0.16	0.08	43.97	4.39	90.01
Yellow knitted waste	50.48	19.44	49.36	79.05	0.97	2.12	69.99	5.09	92.73
Mixed shoddy waste	14.36	2.35	-9.72	77.43	1.60	1.83	69.9	6.64	90.51

The control sample was a new bleached white 50% polyester/50% cotton fabric ready for dyeing.

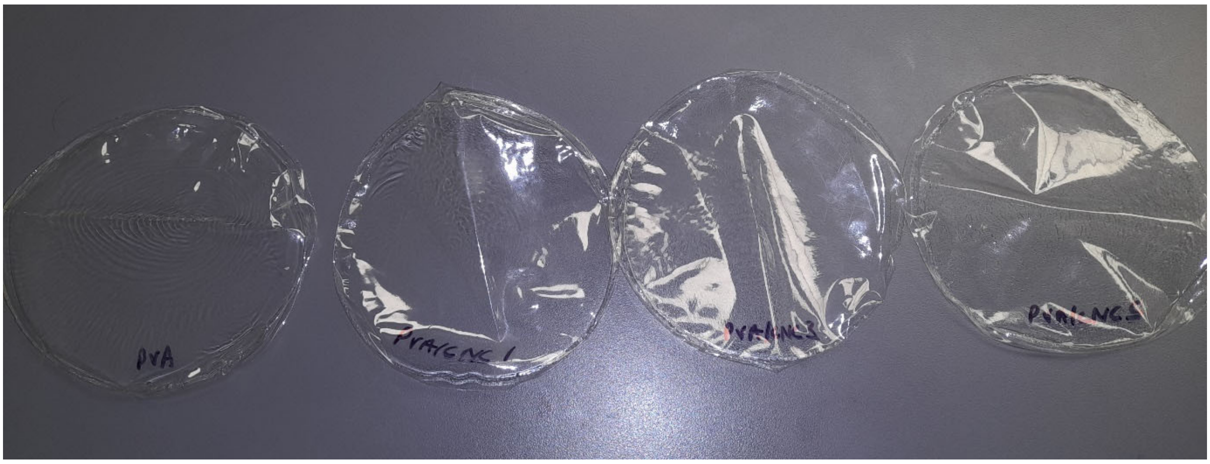

Figure S2. Photographs of CNC reinforced PVA composite films.
